# Burned Flour as a Day Dressing for Ill-Conditioned Wounds, Erysipelas, Etc.

**Published:** 1892-07

**Authors:** 


BURNED FLOUR AS A DRY DRESSING FOR ILL-CONDITIONED WOUNDS, ERYSIPELAS, ETC.—Dr. J. J. Crow, of Carrollton, Ill., writes: "I have lately used common flour burned in a stovepan as a dressing for erysipelas, senile gangrene, and burns, and am much pleased with it. It is almost everywhere present, and may be prepared in a few minutes. It is cheap and may be impregnated with iodoform, aristole, or other antiseptics. I hope the profession will give it a fair trial. It is, of course, finely divided charcoal.—Ex.

